# Effects of dexpanthenol on 5-fluorouraci-induced nephrotoxicity, hepatotoxicity, and intestinal mucositis in rats: a clinical, biochemical, and pathological study

**DOI:** 10.2478/abm-2025-0006

**Published:** 2025-02-28

**Authors:** Şeyma Taştemur, Mehmet Ekĭcĭ, Ali Sefa Mendĭl, Mustafa Özkaraca, Hilmi Ataseven

**Affiliations:** Department of Internal Medicine, Faculty of Medicine, Sivas Cumhuriyet University, Sivas 58140, Turkey; Department of Veterinary Physiology, Faculty of Veterinary Medicine, Sivas Cumhuriyet University, Sivas 58140, Turkey; Department of Veterinary Pathology, Faculty of Veterinary Medicine, Erciyes University, Kayseri 38280, Turkey; Department of Veterinary Pathology, Faculty of Veterinary Medicine, Sivas Cumhuriyet University, Sivas 58140, Turkey; Department of Gastroenterology, Faculty of Medicine, Sivas Cumhuriyet University, Sivas 58140, Turkey

**Keywords:** 5-fluorouracil, dexpanthenol, hepatotoxicity, intestinal mucositis, nephrotoxicity

## Abstract

**Background:**

5-fluorouracil (5-FU) is a broad-spectrum drug that has a wide range of side effects. Patients may experience severe comorbidities as a result of these toxic side effects, making it impossible for them to continue chemotherapy. Despite the fact that various molecules have been experimented, there is no literature data on the efficacy of dexpanthenol (DXP) for mitigating the toxic effects of 5-FU.

**Objective:**

To investigate the protective effects of DXP on nephrotoxicity, hepatotoxicity, and intestinal toxicity induced by 5-FU in rats.

**Methods:**

Twenty-eight male Wistar-Albino rats aged 16 weeks were randomly assigned to four groups. We created a rat model of intestinal mucositis, nephrotoxicity, and hepatotoxicity through intraperitoneal 5-FU (35 mg/kg for 4 d) injection. 500 mg/kg and 1000 mg/kg of DXP were administered to the treatment groups. The effects of dexpanthenol were evaluated clinically, biochemically, histopathologically, and immunohistochemically (inducible nitric oxide synthase [iNOS], cyclooxygenase-2 [COX-2], 8-hydroxyguanosine [8-OHdG], and nuclear factor kappa B [NF-κB]).

**Results:**

5-FU caused a decrease in body weight and food intake, and an increase in diarrhea scores in rats. 5-FU led to significant disruptions in the hepatic biochemical markers (aspartate transaminase [AST], alanine transaminase [ALT], alkaline phosphatase [ALP], total bilirubin, direct bilirubin, and lactate dehydrogenase [LDH]), renal biochemical markers (blood urea nitrogen [BUN], creatinine, and uric acid), and protein and albumin, which are markers of both hepatic and renal functions. Severe pyknosis and mononuclear cell infiltrations were observed in the liver, and mononuclear cell infiltration and tubular degeneration in the kidneys. Jejunum and colon showed villous hyperemia and hemorrhage, respectively, along with mononuclear cell infiltration. Furthermore, 5-FU increased the immunohistochemical expressions of iNOS, COX-2, 8-OHdG, and NF-κB in the examined tissues. The administration of DXP at doses of 500 mg/kg and 1000 mg/kg demonstrated significant mitigation of the toxic effects induced by 5-FU on the liver, kidney, jejunum, and colon.

**Conclusion:**

DXP showed protective effects against nephrotoxicity, hepatotoxicity, and intestinal toxicity caused by 5-FU. These findings suggest that DXP may serve as a potential therapeutic agent to alleviate the severe side effects of 5-FU chemotherapy, thereby improving patient tolerance and quality of life. Further clinical studies are warranted to validate these results and explore the translational potential of DXP in human cancer therapy.

5-fluorouracil (5-FU), a chemotherapeutic medication developed in 1957, disrupts DNA synthesis by inhibiting thymidylate synthase during the S phase of the cell cycle [1, 2]. However, it has a wide range of side effects because 5-FU is a broad-spectrum drug that can operate on healthy tissues and tumor cells. It promotes the release of reactive oxygen species (ROS) and reactive nitrogen species (RNS). The activation of nuclear factor kappa B (NF-κB) and other transcription factors causes the generation of proinflammatory cytokines and inducible nitric oxide synthase (iNOS). Additionally, a stress protein known as cyclooxygenase-2 (COX-2) increases in response to 5-FU and other chemotherapeutics [3,4,5,6,7].

Dexpanthenol (DXP), or provitamin B5, is primarily used in dermatology to reinforce the skin barrier [8]. DXP, a stable alcoholic analog of pantothenic acid, has been clinically used for various skin disorders due to its multiple pharmacological effects. It is found in various skincare products, known particularly for its moisturizing, anti-inflammatory, and wound-healing properties, which make it a promising treatment option for skin disorders [9, 10]. It has been reported that intramuscular injection of DXP at doses of 250 mg and 500 mg in humans has shown positive responses against male androgenetic alopecia, female pattern hair loss, and diffuse hair loss [11,12,13]. In tissues, it is oxidized to vitamin B (pantothenic acid), which increases the synthesis of reduced glutathione (GSH), co-enzyme A (CoA), and cellular adenosine triphosphate (ATP). DXP exhibits anti-inflammatory, antioxidant, and epithelializing properties [14]. DXP's antioxidant properties and ability to stimulate antioxidant systems and reduce inflammation contribute to its protective effects in various tissues and diseases. It reduces oxidative damage by decreasing malondialdehyde (MDA) levels, a marker of lipid peroxidation, attenuates myeloperoxidase (MPO) activity, stimulates the activity of antioxidant enzymes such as superoxide dismutase (SOD) and catalase (CAT), increases the levels of reduced GSH, suppresses apoptosis, and alleviates brain damage. DXP also inhibits the influx of neutrophils and reduces the release of pro-inflammatory cytokines such as TNF-α and IL-6 [15, 16]. In toxicity models in rats, DXP has shown positive effects on the recovery of damaged liver, kidneys, colon, brain, and lung tissues. It has been effective in experimental models such as necrotizing enterocolitis, acetic acid-induced colitis, LPS-induced acute lung and kidney injury, sepsis-related kidney and liver damage, methotrexate-induced liver oxidative toxicity, and traumatic brain injury [15,16,17,18,19,20,21]. Based on the literature review, it was hypothesized that DXP could have positive effects on systemic toxicity induced by 5-FU in this study.

The literature review did not reveal any studies investigating the effect of DXP on systemic toxicity induced by 5-FU. This study aimed to examine the protective effects of DXP on 5-FU-induced hepatotoxicity, nephrotoxicity, and intestinal mucositis in rats.

## Methods

### Animals

This study was conducted at the Experimental Animals Application and Research Center. The principles of the Declaration of Helsinki were followed for all protocols. This study was approved by Sivas Cumhuriyet University's ethics committee (Date: 22.09.2023, Decision: 65202830-050.04.04-764).

Animals were housed in controlled conditions with 22°C–24°C room temperature, 40%–60% humidity, and a 12-h alternating light-dark environment. Animals were provided with feed and tap water *ad libitum*. All efforts were made to minimize animal suffering. Results were reported following the ARRIVE guidelines [22].

### Experimental design

Twenty-eight male Wistar-Albino rats aged 16 weeks weighing 250–300 g were randomly assigned to four groups (n = 7 per group). The control group received 1 mL/kg/d of sterile saline intraperitoneally for 8 d. The 5-FU group received 35 mg/kg/d of 5-FU (Kocak Farma Pharmaceuticals, Istanbul, Turkiye) intraperitoneally for 4 d [23, 24]. The 5-FU+DXP500 group received 35 mg/kg/d of 5-FU and 500 mg/kg/d DXP intraperitoneally for 4 d and 8 d, respectively. The 5-FU+DXP1000 group received 35 mg/kg/d of 5-FU and 1000 mg/kg/d DXP intraperitoneally for 4 d and 8 d, respectively [25]. Previous studies have reported that DXP at doses of 500 mg/kg and 1000 mg/kg exhibits antioxidant, anti-inflammatory, and cytoprotective properties [25, 26]. In this study, we aimed to investigate the effects of increasing doses of DXP (500 mg/kg and 1000 mg/kg).

**Figure 1.**
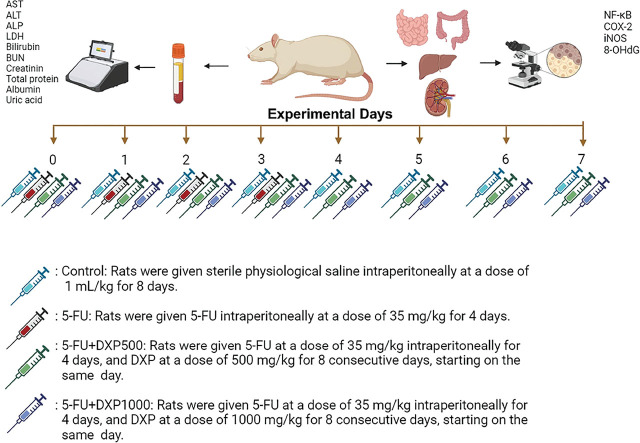
Experimental design. 5-FU, 5-fluorouracil; 8-OHdG, 8-hydroxyguanosine; ALP, alkaline phosphatase; ALT, alanine transaminase; AST, aspartate transaminase; BUN, blood urea nitrogen; COX-2, cyclooxygenase-2; DXP, dexpanthenol; LDH, lactate dehydrogenase; iNOS, inducible nitric oxide synthase; NF-κB, nuclear factor kappa B.

Rats were monitored for changes in food intake, body weight, and diarrhea scores related to mucositis. The diarrhea scores were classified as 0 for no diarrhea, 1 for mild diarrhea (slightly wet, loose stools), 2 for moderate diarrhea (wet, shapeless stools), and 3 for severe diarrhea (watery stools, spotting in the perianal area). Blood samples were collected 24 h after the final drug administration under anesthesia for serum analysis. Serum samples were stored at −80°C for biochemical analyses. Rats were humanely euthanized, and histopathological and immunohistochemical examinations were performed on liver, kidney, jejunum, and colon tissue samples (**Figure 1**).

### Biochemical assessment

At the end of the experiment, the biochemical parameters were analyzed from the serum samples obtained from the rats. Analyzes for renal function tests (blood urea nitrogen [BUN], creatinine, uric acid, total protein, and albumin) and hepatic function tests (aspartate transaminase [AST], alanine transaminase [ALT], alkaline phosphatase [ALP], lactate dehydrogenase [LDH], total bilirubin, and direct bilirubin) were performed using the Roche Cobas 8000 autoanalyzer C702 module (Roche Diagnostics, Mannheim, Germany).

### Histopathological assessment

The rats underwent necropsy, and liver, kidney, jejunum, and colon tissues were preserved in a 10% neutral formalin solution. After routine alcohol-xylene follow-up procedures, the tissues were embedded in paraffin blocks. Sections of 5 μm on poly-lysine slides were stained with hematoxylin–eosin and semi-quantitatively evaluated for degenerative, necrotic-pyknotic, and inflammatory changes, categorized as absent (−), mild (+), moderate (++), or severe (+++) in five different microscope fields according to **Table 1**.

**Table 1.** Histopathological scoring

Liver	Degenerative, necrotic-pyknotic hepatocytes in microscope fields;
	Absent (−), 1–5 < (+, mild), 6–9 (++, moderate), 9> (+++, severe)
	Infiltrative mononuclear cell count in microscope fields;
	Absent (−), 2–10 < (+, mild), 11–20 (++, moderate), 21> (+++, severe)

Kidney	Degenerative tubule in microscope fields;
	Absent (−), 1–5 < (+, mild), 6–10 (++, moderate), 11> (+++, severe)
	Mononuclear cell focus in microscope fields;
	Absent (−), 1 (+, mild), 2 (++, moderate), 3> (+++, severe)

Jejenum and colon	Hypremia focus in microscope fields;
	Absent (−), 1–5 (+, mild), 6–10 (++, moderate), 11> (+++, severe)
	Area of the villus (%) in microscope fields;
	Absent (−), 25 < (+, mild), 26–50 (++, moderate), 51> (+++, severe)

### Immunohistochemical assessment

Five micrometer-thick tissue sections on poly-lysine slides were deparaffinized using xylene and alcohol series, followed by a PBS wash. Endogenous peroxidase activity was inhibited by treating tissues with 3% H_2_O_2_ for 10 min. Antigen retrieval involved two rounds of 5-min treatments with antigen retrieval solution at 500 watts. The tissues were incubated overnight at 4°C with primary antibodies (8-hydroxyguanosine [8-OHdG] from Santa Cruz, Cat. no. sc-66036; NF-κB from Abcam, Cat. no. ab7971; COX-2 from Abcam, Cat. no. ab-15191; and iNOS from Bioss, Cat. no. bs-2072R) at a 1/200 dilution. For secondary detection, the Large Volume Detection System: anti-Polyvalent, HRP (ThermoFisher, Catalog no: TP-125-HL) was used as per the manufacturer's guidelines. Chromogen staining utilized 3-amino-9-ethylcarbazole (AEC). The slides were examined under a light microscope following counterstaining with Mayer's Hematoxylin. Immunopositivity was evaluated as absent (−), mild (+), moderate (++), severe (+++), and very severe (++++) based on the examination of five different microscope fields according to **Table 2**.

**Table 2.** Immunohistochemical scoring

Liver	Immunopositivity cells count
	Absent (−), 5 < (+, mild), 5–10 (++, moderate), 10> (+++, severe)

Kidney	Immunopositivity tubules count
	Absent (−), 5 < (+, mild), 5–10 (++, moderate), 10> (+++, severe)

Jejenum and colon	Area of the villus (%)
	Absent (−), 5 < (+, mild), 5–15 (++, moderate), 15–30> (+++, severe)
	30%> (++++, very severe)

### Statistical analysis

The sample size of n = 7 per group was determined using G*Power Version 3.1.9.6 (Germany) software based on previous study data [21]. The effect size, α 10 error probability, and power (1-β error probability) were set at 0.79, 0.05, and 0.90, respectively. Clinical and biochemical data were analyzed using one-way ANOVA and *post hoc* Tukey test in GraphPad Prism 8.0.1 software. Histopathological data were analyzed using SPSS 20.00 software. Kruskal–Wallis test and Mann–Whitney *U* test (*P* < 0.05) were used to determine the differences between groups. Results were presented as mean ± standard error of the mean (SEM). Statistical significance was considered at *P* < 0.05.

## Results

### Clinical findings

**Figure 2.**
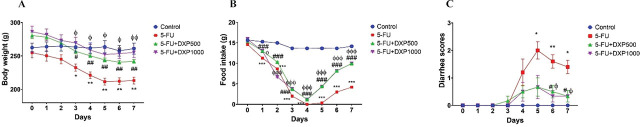
**(A)** body weight changes, (**B**) food intake changes, and (**C**) diarrhea score changes. ^*^P < 0.05, ^**^P < 0.01, and ^***^P < 0.001 are significant 5-FU group compared with control group; ^#^*P* < 0.05, ^##<^*I*>*P* < 0.01, and ^###^*P* < 0.001 are significant 5-FU+DXP500 group compared with 5-FU group; ^ϕ^*P* < 0.05, ^ϕϕ^*P* < 0.01, and ^ϕϕϕ^*P* < 0.001 are significant 5-FU+DXP1000 group compared with 5-FU group according to one-way ANOVA *post hoc* Tukey test in Graph Pad Prism 8.0.1 software. 5-FU, 5-fluorouracil; DXP, dexpanthenol.

Statistically significant weight loss was observed in the 5-FU group compared with the control group, 5-FU+DXP500 group, and 5-FU+DXP1000 group after 8 d (*P* < 0.01) (**Figure 2A**). Administration of DXP at 500 mg/kg and 1000 mg/kg decreased the rate of weight loss in the 5-FU+DXP500 and 5-FU+DXP1000 groups even turning the curves into a positive trend at the end of the test. Although the 5-FU+DXP500 and 5-FU+DXP1000 groups did not regain their previous mass, the weight loss in the 5-FU+DXP500 and 5-FU+DXP1000 groups was not as significant as in the 5-FU group. Daily food intake decreased significantly in the 5-FU group at the end of the study (*P* < 0.001).

The decrease in daily food intake observed in both 5-FU+DXP500 and 5-FU+DXP1000 groups continued until day 6. However, from day 6 onward, there was a significant increase in food intake in these groups. This increase, attributed to DXP administration, resulted in a statistically significantly more pronounced decrease in food intake in the 5-FU group compared with the 5-FU+DXP500 and 5-FU+DXP1000 groups (*P* < 0.001) (**Figure 2B**).

Rats were monitored for diarrhea that would correlate with mucositis. The 5-FU group had notably increased diarrhea scores on days 5, 6, and 7 compared with the control group (*P* < 0.05, *P* < 0.01, *P* < 0.05, respectively). Diarrhea scores were significantly lower in the 5-FU+DXP500 and 5-FU+DXP1000 groups compared with the 5-FU group (*P* < 0.05) (**Figure 2C**).

### Biochemical findings

**Figure 3.**
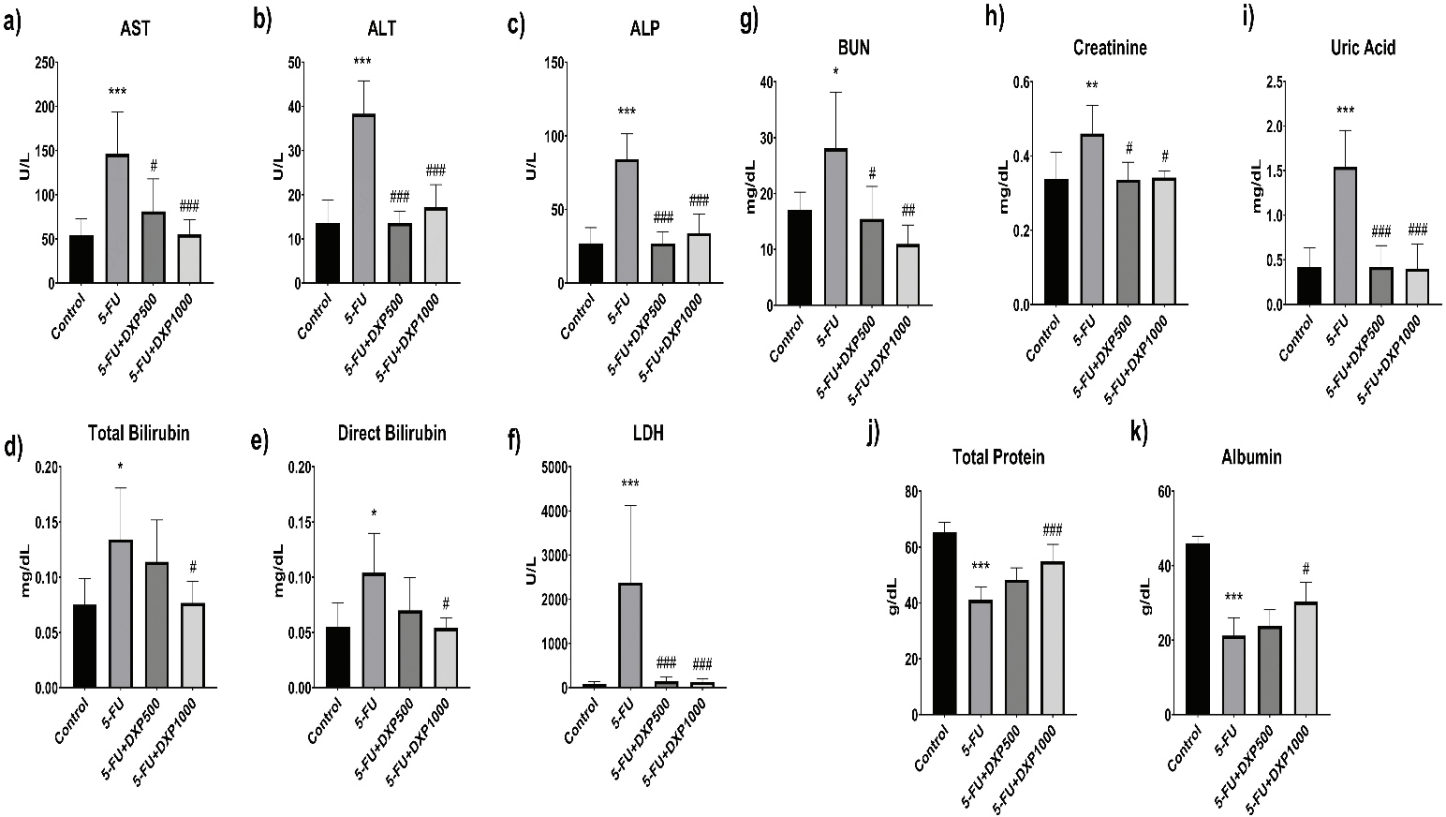
Liver and renal biochemical analysis results. AST (**a**), ALT (**b**), ALP (**c**), total bilirubin (**d**), direct bilirubin (**e**), LDH (**f**), BUN (**g**), creatinine (**h**), uric acid (**i**), total protein (**j**), and albumin (**k**) level changes in groups. ^*^P < 0.05, ^**^P < 0.01, and ^***^P < 0.001 are significant 5-FU group compared with the control group; ^#^*P* < 0.05, ^##^*P* < 0.01, and ^###^*P* < 0.001 are significant 5-FU+DXP500 and 5-FU+DXP1000 groups compared with 5-FU group according to one-way ANOVA *post hoc* Tukey test in Graph Pad Prism 8.0.1 software. 5-FU, 5-fluorouracil; ALP, alkaline phosphatase; ALT, alanine transaminase; AST, aspartate transaminase; BUN, blood urea nitrogen; DXP, dexpanthenol; LDH, lactate dehydrogenase.

Statistically significant impairment in hepatic and renal function tests was observed in 5-FU-treated rats compared to the control group. 5-FU caused a significant increase in AST (*P* < 0.001), ALT (*P* < 0.001), ALP (*P* < 0.001), LDH (*P* < 0.001), total bilirubin (*P* < 0.05), and direct bilirubin (*P* < 0.05) levels in rats (**Figure 3**). When DXP was given with 5-FU at 1000 mg/kg doses, all parameters decreased significantly, especially AST, ALT, ALP, and LDH levels (*P* < 0.001). The 500 mg/kg DXP dose did not positively affect total bilirubin and direct bilirubin levels (*P* > 0.05), but it did lead to a significant decrease in ALT (*P* < 0.001), ALP (*P* < 0.001), LDH (*P* < 0.001), and AST (*P* < 0.05).

5-FU caused significant changes in BUN (*P* < 0.05), creatinine (*P* < 0.01), uric acid (*P* < 0.001), total protein (*P* < 0.001), and albumin (*P* < 0.001) levels in rats (**Figure 3**). Administration of DXP with 500 mg/kg 5-FU resulted in a highly significant decrease in uric acid levels (*P* < 0.001) and a significant decrease in BUN and creatinine levels (*P* < 0.05).

Five hundered milligram per kilogram of 5-FU did not have a significant positive effect on total protein and albumin levels. Thousand milligram per kilogram of 5-FU administration showed significant improvement in both total protein (*P* < 0.001) and albumin (*P* < 0.05) levels.

### Histopathologic findings

The liver, kidney, jejunum, and colon of the control group rats had normal histologic appearance. Histopathologic examination revealed statistically significant differences between the groups in the liver, kidney, jejunum, and colon samples (**Table 3**, *P* < 0.05).

**Table 3.** Histopathological findings and statistical analysis of immunohistochemical staining in liver, renal, jejunum, and colon tissues

**a) Histopathological findings and statistical analysis of immunohistochemical staining in liver tissues**							

**Groups**	**Mononuclear cell infiltration**	**Pyknosis in hepatocytes**	**Degeneration in hepatocytes**	**8-OHdG**	**NF-κB**	**COX-2**	**iNOS**

Control	0.16 ± 0.40^†^	0.33 ± 0.51^†^	0.33 ± 0.51^†^	0.00 ± 0.00^†^	0.00 ± 0.00^†^	0.00 ± 0.00^†^	0.00 ± 0.00^†^
5-FU	2.83 ± 0.40^‡^	2.66 ± 0.51^‡^	1.00 ± 0.00^‡^	2.66 ± 0.51^†^	2.83 ± 0.40^‡^	1.83 ± 0.40^‡^	2.66 ± 0.51^‡^
5-FU+DXP500	2.00 ± 0.00^§^	1.83 ± 0.40^§^	1.00 ± 0.00^‡^	1.66 ± 0.51^§^	1.83 ± 0.40^§^	1.66 ± 0.51^‡^	1.66 ± 0.51^§^
5-FU+DXP1000	1.00 ± 0.00^‖^	1.33 ± 0.51^‖^	1.16 ± 0.40^‡^	0.83 ± 0.40^‖^	1.00 ± 0.00^‖^	0.66 ± 0.51^‖^	0.66 ± 0.51^‖^

**b) Histopathological findings and statistical analysis of immunohistochemical staining in renal tissues**							

**Groups**	**Mononuclear cell infiltration**	**Tubular degeneration**		**8-OHdG**	**NF-κB**	**COX-2**	**iNOS**

Control	0.16 ± 0.40^†^	0.33 ± 0.51^†^		0.00 ± 0.00^†^	0.00 ± 0.00^†^	0.83 ± 0.40^†^	0.83 ± 0.40^†^
5-FU	1.83 ± 0.40^‡^	2.00 ± 0.00^‡^		0.83 ± 0.40^‡^	0.66 ± 0.51^‡^	2.83 ± 0.40^‡^	2.66 ± 0.51^‡^
5-FU+DXP500	0.83 ± 0.40^§^	1.00 ± 0.00^§^		0.00 ± 0.00^†^	0.00 ± 0.00^†^	1.66 ± 0.51^§^	1.83 ± 0.40^§^
5-FU+DXP1000	1.00 ± 0.00^§^	0.83 ± 0.40^§^		0.00 ± 0.00^†^	0.00 ± 0.00^†^	1.83 ± 0.40^§^	1.83 ± 0.40^§^

**c) Histopathological findings and statistical analysis of immunohistochemical staining in jejunum tissues**							

**Groups**	**Mononuclear cell infiltration**	**Villous hyperemia**		**8-OhdG**	**NF-κB**	**COX-2**	**iNOS**

Control	0.00 ± 0.00^†^	0.00 ± 0.00^†^		0.33 ± 0.51^†^	0.33 ± 0.51^†^	1.00 ± 0.00^†^	1.16 ± 0.40^†^
5-FU	2.16 ± 0.40^‡^	1.66 ± 0.51^‡^		1.66 ± 0.51^‡^	2.66 ± 0.51^‡^	2.83 ± 0.40^‡^	3.66 ± 0.51^‡^
5-FU+DXP500	1.00 ± 0.00^§^	0.83 ± 0.40^§^		0.66 ± 0.51^§^	1.83 ± 0.40^§^	1.83 ± 0.40^§^	2.83 ± 0.40^§^
5-FU+DXP1000	1.00 ± 0.00^§^	0.66 ± 0.51^§^		0.66 ± 0.51^§^	1.66 ± 0.51^§^	2.00 ± 0.00^§^	1.66 ± 0.51^§^

**d) Histopathological findings and statistical analysis of immunohistochemical staining in colonic tissues**							

**Groups**	**Mononuclear cell infiltration**	**Hemorrhage**		**8-OhdG**	**NF-κB**	**COX-2**	**iNOS**

Control	0.00 ± 0.00^†^	0.00 ± 0.00^†^		0.66 ± 0.51^†^	0.16 ± 0.40^†^	1.16 ± 0.40^†^	0.66 ± 0.40^†^
5-FU	0.66 ± 0.51^‡^	1.66 ± 0.51^‡^		2.83 ± 0.40^‡^	1.66 ± 0.51^‡^	3.00 ± 0.00^‡^	1.66 ± 0.51^‡^
5-FU+DXP500	0.00 ± 0.00^†^	0.66 ± 0.51^§^		2.00 ± 0.00^§^	0.66 ± 0.51^§^	2.16 ± 0.40^§^	0.66 ± 0.51^†^
5-FU+DXP1000	0.16 ± 0.40^†^	0.66 ± 0.51^§^		2.16 ± 0.40^§^	0.66 ± 0.51^§^	2.16 ± 0.40^§^	0.83 ± 0.40^†^

^†, ‡, §, ‖^indicate the difference between groups, *P* < 0.05.

^†^*P* < 0.05: Difference between the control group and the other groups.

^‡^*P* < 0.05: Difference between the 5-FU group and the other groups.

^§^*P* < 0.05: Difference between the 5-FU+DXP500 group and the other groups.

^‖^*P* < 0.05: Difference between the 5-FU+DXP1000 group and the other groups.

5-FU, 5-fluorouracil; 8-OhdG, 8-hydroxyguanosine; COX-2, cyclooxygenase-2; DXP, dexpanthenol; iNOS, inducible nitric oxide synthase; NF-κB, nuclear factor kappa B.

**Figure 4.**
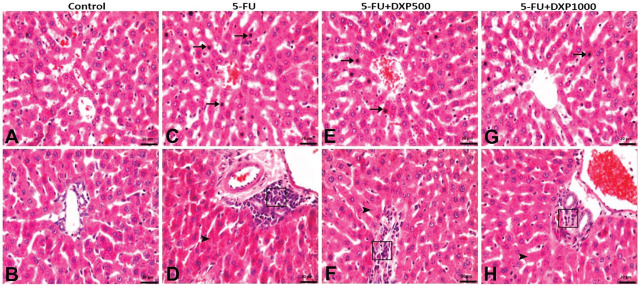
Images of histopathologic findings in H&E stained liver tissues. (**A, B**) Control group: Normal histologic appearance. (**C, D**) 5-FU group: Severe hepatocyte pyknosis (arrow), mononuclear cell infiltration around the bile ducts (□), and mild hepatocyte degeneration (arrowhead). (**E, F**) 5-FU+DXP500 group: Mononuclear cell infiltrations around the bile ducts (□) with moderate hepatocyte pyknosis (arrow) and mild hepatocyte degeneration (arrowhead). (**G, H**) 5-FU+DXP1000 group: Mild hepatocyte pyknosis (arrow), mononuclear cell infiltrations around the bile ducts (□), and hepatocyte degeneration (arrowhead). 5-FU, 5-fluorouracil; DXP, dexpanthenol.

Degeneration and pyknosis in hepatocytes and mononuclear cell infiltrations in periportal areas were detected in the liver of the treatment groups. Among these findings, degeneration of hepatocytes was mild in all treatment groups. Pyknosis and mononuclear cell infiltrations were severe in the 5-FU group, moderate in the 5-FU+DXP500 group, and mild in the 5-FU+DXP1000 group (**Table 3a** and **Figure 4**).

**Figure 5.**
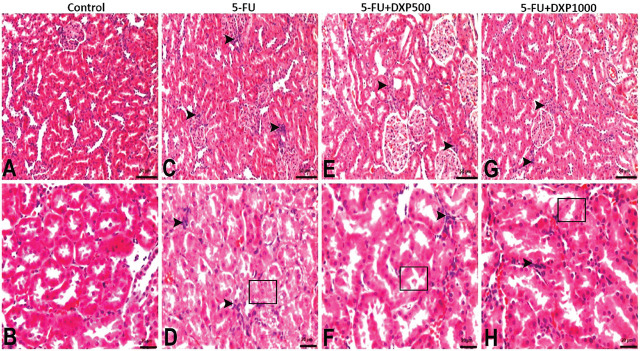
Images of histopathologic findings in H&E stained renal tissues. (**A, B**) Control group: Normal histologic appearance. (**C, D**) 5-FU group: Moderate mononuclear cell infiltration in interstitial areas (arrowhead) and degenerative tubules (□). (**E, F**) 5-FU+DXP500 group and (**G, H**) 5-FU+DXP1000 group: Mild mononuclear cell infiltrations (arrowhead) and degenerative tubules (□) in interstitial areas. 5-FU, 5-fluorouracil; DXP, dexpanthenol.

Mononuclear cell infiltrations in the renal interstitial areas and tubular degenerations were moderate in the 5-FU group and mild in the 5-FU+DXP500 and 5-FU+DXP1000 groups (**Table 3b** and **Figure 5**).

**Figure 6.**
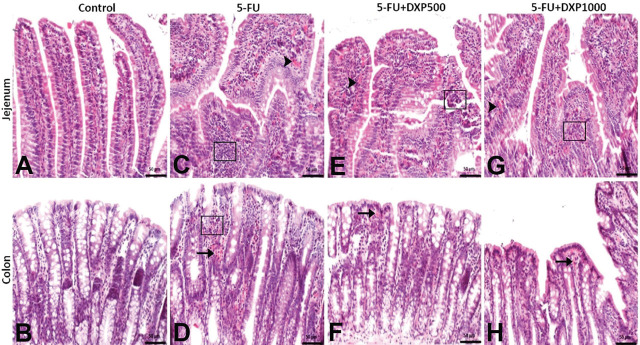
Images of histopathologic findings in H&E stained jejunum and colon tissues. (**A, B**) Control group: Normal histologic appearance. (**C, D**) 5-FU group: Moderate hyperemia (arrowhead), mononuclear cell infiltrations (□), moderate hemorrhage (arrow), and mild mononuclear cell infiltrations (□) in the jejunum. (**E, F**) 5-FU+DXP500 group and (**G, H**) 5-FU+DXP1000 group: Mild hyperemia (arrowhead), mononuclear cell infiltrations (□), and hemorrhage (arrow) in the jejunum and colon. 5-FU, 5-fluorouracil; DXP, dexpanthenol.

The histopathologic findings observed on examinations of intestine/colon tissues were hyperemia in the villi of the jejunum, subepithelial hemorrhage areas in the colonic mucosa, and mononuclear cell infiltrations in the jejunum and colon (**Figure 6**). In the jejunum, villous hyperemia and mononuclear cell infiltration were moderate in the 5-FU group, whereas mild in the 5-FU+DXP500 and 5-FU+DXP1000 groups. Colonic mucosal hemorrhage was found to be moderate in the 5-FU group and mild in the 5-FU+DXP500 and 5-FU+DXP1000 groups. In addition, colonic mononuclear cell infiltration, which was mild in the 5-FU group, was not observed in the other treatment groups (**Tables 3c,d** and **Figure 6**).

### Immunohistochemical expressions of 8-OHdG, NF-κB, COX-2, and iNOS in the liver, kidney, jejunum, and colon tissues

**Figure 7.**
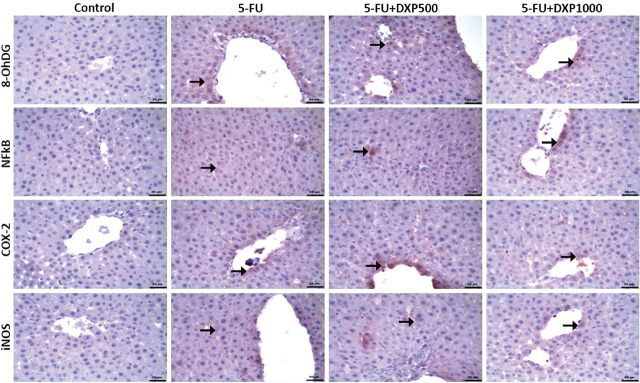
Images of immunohistochemical findings in liver tissues. Severe immunopositivity in the 5-FU group, moderate immunopositivity in the 5-FU+DXP500 group, and mild immunopositivity in the 5-FU+DXP1000 group for 8-OHdG, NF-κB, COX-2, and iNOS (arrows), respectively. 5-FU, 5-fluorouracil; 8-OHdG, 8-hydroxyguanosine; COX-2, cyclooxygenase-2; DXP, dexpanthenol; iNOS, inducible nitric oxide synthase; NF-κB, nuclear factor kappa B.

**Figure 8.**
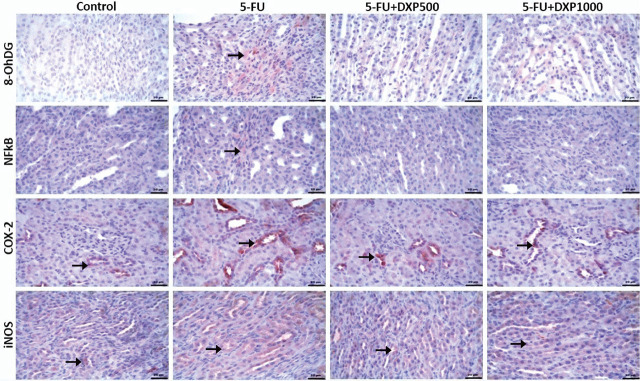
Images of immunohistochemical findings in renal tissues. In the control group, there was mild immunopositivity for COX-2 and iNOS, while in the 5-FU group, there was mild immunopositivity for 8-OHdG and NF-κB, and severe immunopositivity for COX-2 and iNOS. In the 5-FU+DXP500 and 5-FU+DXP1000 groups, there was moderate immunopositivity for COX-2 and iNOS (arrows). 5-FU, 5-fluorouracil; 8-OHdG, 8-hydroxyguanosine; COX-2, cyclooxygenase-2; DXP, dexpanthenol; iNOS, inducible nitric oxide synthase; NF-κB, nuclear factor kappa B.

**Figure 9.**
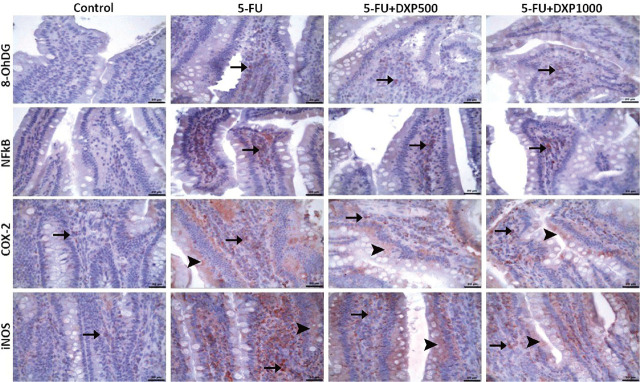
Images of immunohistochemical findings in jejunum tissues. Mild COX-2 and iNOS immunopositivity in the control group, moderate 8-OHdG, severe NF-κB, COX-2, and very severe iNOS immunopositivity in the 5-FU group, mild 8-OHdG, moderate NF-κB, COX-2, and severe iNOS immunopositivity in the 5-FU+DXP500 and 5-FU+DXP100 groups (arrows: immunopositivity in the villi, arrowheads: immunopositivity in the lamina epithelialis). 5-FU, 5-fluorouracil; 8-OHdG, 8-hydroxyguanosine; COX-2, cyclooxygenase-2; DXP, dexpanthenol; iNOS, inducible nitric oxide synthase; NF-κB, nuclear factor kappa B.

**Figure 10.**
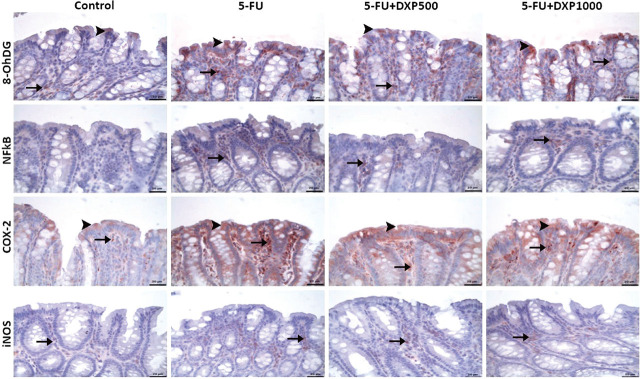
Images of immunohistochemical findings in colonic tissues. Mild 8-OHdG, COX-2 and iNOS immunopositivity in the control group, severe 8-OHdG, moderate NF-κB, severe COX-2, and moderate iNOS immunopositivity in the 5-FU group, moderate 8-OHdG, mild NF-κB, moderate COX-2, and mild iNOS immunopositivity in the 5-FU+DXP500 and 5-FU+DXP100 groups (arrows: immunopositivity in the mucosa, arrowheads: immunopositivity in the lamina epithelialis). 5-FU, 5-fluorouracil; 8-OHdG, 8-hydroxyguanosine; COX-2, cyclooxygenase-2; DXP, dexpanthenol; iNOS, inducible nitric oxide synthase; NF-κB, nuclear factor kappa B.

There were statistically significant differences between the groups regarding 8-OHdG, NF-κB, COX-2, and iNOS staining in the liver, kidney, jejunum, and colon samples (**Table 3**, *P* < 0.05). The immunopositivity of 8-OHdG, NF-κB, COX-2, and iNOS in the liver tissues was severe in the 5-FU group, moderate in the 5-FU+DPX500 group, and mild in the 5-FU+DPX1000 group (**Table 3a** and **Figure 7**). Immunohistochemical assessment of renal tissues showed mild 8-OHdG and NF-κB immunopositivity in the 5-FU group. No significant immunopositivity was found in the control and other treatment groups. COX-2 and iNOS immunopositivity were mild in the control group, severe in the 5-FU group, and moderate in the 5-FU+DXP500 and 5-FU+DXP1000 groups (**Table 3b** and **Figure 8**). Immunohistochemical staining of the jejunum and colon tissues revealed that immunopositivity for 8-OHdG, NF-κB, COX-2, and iNOS, which were generally high, decreased in 5-FU+DXP500 and 5-FU+DXP1000 groups (**Tables 3c,d** and **Figures 9 and 10**). No significant difference was observed between 5-FU+DXP500 and 5-FU+DXP1000 groups.

## Discussion

Chemotherapy medications might cause serious negative effects. Cancer research requires developing techniques to improve chemotherapy effectiveness and lessen side effects. This study focused on DXP's preventive impact against the toxic effects of 5-FU. DXP improved 5-FU-induced nephrotoxicity, hepatotoxicity, and intestinal mucositis in rats. The results suggest that DXP could be a promising therapeutic for mitigating the side effects of 5-FU chemotherapy.

Rats treated with 5-FU experienced decreased food intake, weight loss, and increased diarrhea scores as in similar studies [27, 28]. DXP showed ameliorative effects on hepatic, renal, and intestinal injury in rats. Weight loss and decreased food intake are associated with nephrotoxicity and hepatotoxicity, while diarrhea scoring is more specific for intestinal mucositis. 500 mg/kg/d and 1000 mg/kg/d of DXP significantly improved observational metabolic data in the 5-FU-induced intestinal mucositis model. DXP reduced the systemic effects of 5-FU-induced inflammation and intestinal mucositis in rats, suggesting that it could potentially alleviate constitutional symptoms in cancer patients.

In the studies, the effects of various molecules on 5-FU-induced nephrotoxicity and hepatotoxicity were examined using serum samples from rats [29, 30]. In this study, we performed biochemical renal and liver function tests including ALT, AST, ALP, total bilirubin, direct bilirubin, LDH, BUN, creatinine, uric acid, total protein, and albumin levels in rats. These tests showed that 5-FU impaired hepatic and renal function. DXP showed an ameliorating effect on this dysfunction with a decrease in BUN, creatinine, and uric acid levels and an increase in total protein and albumin levels. Prevention of renal toxicity in cancer patients receiving 5-FU is very important due to the severe and irreversible nature of renal injury resulting in chronic renal failure. 5-FU negatively affected liver functions by increasing ALT, AST, ALP, total bilirubin, direct bilirubin, and LDH levels, and decreasing total protein and albumin levels. DXP administration decreased significantly ALT and AST the markers of hepatocellular damage and ALP the marker of cholestasis, total bilirubin, and direct bilirubin the markers of biliary pathologies. DXP administration in rats ameliorated 5-FU-associated hepatic toxicity, showing promise for cancer patients. High LDH levels indicate tissue damage [31]. In this study, high levels were associated with liver damage, kidney damage, and intestinal mucositis.

Total protein and albumin levels are indicators of liver synthesis function and kidney filtration ability. Albumin, synthesized in the liver, plays a crucial role in bodily functions [32]. Decreased serum albumin levels can indicate nephrotoxicity due to tubular damage causing albumin excretion and hepatotoxicity due to hepatocyte damage. Albumin level is a nutritional indicator and decreased levels are linked to malnutrition [33]. Albumin has 2.5–3 d half-life in rats, allowing for short-term assessment of hepatotoxicity, renal toxicity, and malnutrition. Protein synthesis is also suppressed by inflammatory stimuli like 5-FU, leading to decreased albumin levels. Inflammation accompanied by malnutrition in rats can make it unclear. The factor is more indicative of hypoalbuminemia. Albumin decreases during inflammation as an acute phase response [34]. Therefore, it is not clear which of the two is more indicative of hypoalbuminemia when inflammation is accompanied by malnutrition in rats. However, it is obvious that albumin levels decreased with 5-FU administration. Total protein and albumin levels increase significantly after DXP infusions. This result is valuable when combined with the existing literature data and this study's histopathologic and immunohistochemical findings.

5-FU inhibits DNA replication via thymidylate synthase, leading to DNA damage in tumor cells and rapidly proliferating cells [35]. 5-FU causes intestinal mucositis characterized by villi and crypt atrophy [36]. DXP reduces the histological damage score of colitis by increasing antioxidant capacity. DXP alleviated inflammatory cell infiltration, ulceration, goblet cell depletion, and crypt atrophy in this study [18]. In an animal experiment investigating the effects of silymarin on 5-FU-associated gastrointestinal toxicity, villous hyperemia was observed in the intestines, similar to this study [37]. In this study, we histopathologically examined rats' jejunum and colon tissues to evaluate intestinal toxicity. In addition to mononuclear cell infiltration observed in both jejunum and colon tissues, we found villous hyperemia in jejunum and subepithelial hemorrhage foci in the colon. Symptoms such as diarrhea, nausea, vomiting, and oral mucositis are known to be related to the mucosal damage caused by the cytotoxic effects of 5-FU [5]. Chemotherapy-induced harm to the intestinal mucosa is associated with microinflammation within the mucosal layer. Inflammatory agents can harm endothelial cells, increase local vascular permeability, and stimulate macrophage migration. This process can lead to the production of COX-2 and prostaglandins, resulting in pathological changes like intestinal edema, ulcer formation, and reduced absorption capabilities [38]. Like the effect of silymarin in the study of Safarpour et al. [37], DXP caused a decrease in intestinal histopathologic findings.

Nephrotoxicity due to 5-FU is associated with DNA damage, apoptotic cell death, and increased caspase-3 activity [4, 39]. Excessive accumulation of free oxygen radicals and decreased antioxidant enzyme activity contribute to cytotoxicity. Animal experiments have shown increased ROS [40] and decreased antioxidant enzyme activity [41] with 5-FU. Proinflammatory cytokines like IL-1, IL-6, and TNF-α are elevated in rats administered 5-FU, indicating an inflammatory response mechanism for nephrotoxicity [39]. Histopathological damage caused by 5-FU in the kidneys includes glomerular atrophy, tubular necrosis, dilatation in the bowman capsule and the tubules, and interstitial congestion [29, 42]. Mononuclear cell infiltration and tubular degeneration are detected in the interstitial areas of renal tissue in this study. DXP administration reduced hyperemia, hemorrhage, and tubular degeneration in a lipopolysaccharide-induced acute kidney injury model study, as in the 5-FU nephrotoxicity model in this study [21]. This is important to determine the effect of DXP on renal damage, regardless of the etiology.

5-FU is metabolized in the liver via dihydropyrimidine dehydrogenase. Only a small portion of 5-FU is excreted through the kidneys. 5-FU leads to excessive production of ROS/RNS causing oxidative damage, resulting in a decrease in plasma antioxidant levels. 5-FU-induced hepatotoxicity is probably due to intrinsic hepatotoxicity resulting from thymidylate synthase inhibition. 5-FU is metabolized in the liver via the microsomal enzyme system, producing toxic intermediates that can trigger liver injury [6]. Hepatotoxic effects of 5-FU in rats include the dissolution of hepatic cords, inflammatory cells, necrotic tissues, periportal fibrosis, degeneration of hepatic cords, vacuolar degeneration, and apoptotic cell death [43]. DXP has been found to reduce the hepatotoxic effects of cisplatin in rats by decreasing Kupffer cells and glycogen loss in hepatocytes [26]. In this study, DXP was observed to lessen hepatocyte degeneration and pyknosis caused by 5FU toxicity and reduced mononuclear cell infiltration around periportal and bile ducts. Hepatic structural deterioration was found along with elevated ALT and AST levels. Inflammation around the bile ducts was observed, consistent with elevated ALP, total bilirubin, and direct bilirubin levels. Improvement in 5-FU-induced biliary involvement with DXP treatment has not been clearly documented in previous literature. The results suggest the potential of testing DXP in biliary pathologies.

We immunohistochemically evaluated the effects of DXP on 5-FU-induced renal, hepatic, and intestinal toxicity by comparing the levels of NF-κB, COX-2, iNOS, and 8-OHdG molecules. In terms of these parameters, a significantly higher immunopositivity was detected in the 5-FU group, whereas this immunopositivity decreased in the DXP-treated groups.

NF-κB regulates genes involved in immunity, inflammation, cell proliferation, and cell death. ROS-induced oxidative stress activates NF-κB protein, leading to cellular damage [44]. Excessive NF-κB activity can lead to inflammation and increased ROS generation, despite its role in maintaining oxidant/antioxidant equilibrium [45]. Nfr2 is a transcription factor that regulates the expression of genes for antioxidant and detoxification enzymes. Nfr2 and NF-κB collaborate to maintain redox homeostasis. Nfr2 activation reduces COX-2, iNOS, and TNF-α production, affecting NF-κB activity [46]. An animal study found that antioxidant vitamin C boosted Nfr2 levels and lowered NF-κB levels in a 5-FU-induced hepatotoxicity model [47].

iNOS enables the synthesis of nitric oxide (NO) from L-arginine. iNOS has an active part in immune and inflammatory processes. Over- or irregularly expressed iNOS is linked to various diseases, including sepsis, cancer, neurodegeneration, and pain [48]. Activation of iNOS by inflammatory stimuli leads to increased production of inflammatory cytokines, indirectly activating the NF-κB pathway [49]. Moringa oleifera oil administration decreased high iNOS and NO levels in an animal model of 5-FU-induced nephrotoxicity [50].

Inducible COX-2 is a critical molecule in the onset of inflammation by converting arachidonic acid to prostaglandins [51]. COX-2 is not usually expressed in cells but is highly active during inflammation. NF-κB modulates COX-2 expression [52]. In an animal model, 5-FU-induced intestinal mucositis increases the expression of NF-κB and COX-2 [53]. COX-2 levels were found to be elevated in colon cancer patients switch poor prognosis. Celecoxib, a COX-2 inhibitor, enhanced the chemosensitivity of 5-FU in patients with colon cancer [54].

8-OHdG is a key indication of free radical-induced oxidative DNA damage and thus a biomarker for oxidative stress and carcinogenesis [55]. In the 5-FU-induced nephrotoxicity model, 8-OHdG level increased with 5-FU administration and decreased significantly with added hesperidin and curcumin [29].

Oxidative stress and NF-κB pathway activation play significant roles in 5-FU-induced hepatotoxicity, nephrotoxicity, and intestinal mucositis. DXP can prevent oxidative stress by increasing pro-oxidant molecules, stabilizing cell membranes, and promoting tissue regeneration. In a mice model, DXP reduced inflammation in the liver and kidneys by lowering inflammatory cytokine levels [19]. In a rat model of nicotine-induced liver toxicity, DXP prevented cell death by reducing TOS, inflammatory cytokines, and NF-κB levels through Nrf2 activation, thus protecting against tissue damage [56].

## Conclusion

DXP inhibits 5-FU-induced hepatotoxicity, nephrotoxicity, and intestinal mucositis in rats. This study used clinical, biochemical, histological, and immunohistochemical investigations to show that DXP had a positive effect on liver, kidney, and intestinal tissues. 5-FU induced weight loss, diarrhea, and decreased food intake in the research animals. DXP's clinically benefits on these rats have shown promise for cancer patients by lowering 5-FU-related side effects, which can be difficult to manage and disrupt treatment. DXP could be used to treat various oxidative hepatic, renal, and intestinal illnesses regardless of cause. Further studies are needed to explore the potential use of DXP for this purpose in cancer patients, and the treatment or rescue strategy after distant organ damage caused by 5-FU has occurred.
